# Correction of radiographic measurements of acetabular cup wear for variations in pelvis orientation

**DOI:** 10.1177/0954411918754924

**Published:** 2018-02-23

**Authors:** Brian Derbyshire

**Affiliations:** Centre for Hip Surgery, Wrightington Hospital, Wigan, UK

**Keywords:** Hip prosthesis, total hip replacement, acetabular cup wear, radiographic measurement, pelvic tilt, pelvic orientation, wear correction, wear measurement

## Abstract

Radiographic measurement of two-dimensional acetabular cup wear is usually carried out on a series of follow-up radiographs of the patient’s pelvis. Since the orientation of the pelvis might not be consistent at every X-ray examination, the resulting change in view of the wear plane introduces error into the linear wear measurement. This effect is amplified on some designs of cup in which the centre of the socket is several millimetres below the centre of the cup or circular wire marker. This study describes the formulation of a mathematical method to correct radiographic wear measurements for changes in pelvis orientation. A mathematical simulation of changes in cup orientation and wear vectors caused by pelvic tilt was used to confirm that the formulae corrected the wear exactly if the radiographic plane of the reference radiograph was parallel to the true plane of wear. An error analysis showed that even when the true wear plane was not parallel to the reference radiographic plane, the formulae could still provide a useful correction. A published correction formula was found to be ineffective.

## Introduction

Total hip replacements that have polyethylene (PE) acetabular cups or liners can fail due to a local cellular reaction elicited by wear-generated, microscopic particles. Osteolysis and aseptic loosening of the cup can then ensue. It is widely believed that this reaction is dose dependent: a threshold volume of wear must be exceeded before a reaction is triggered.^1–4^ Much research has therefore focused on assessing the tribological properties of different material combinations for the articulating components of the joint.^5^ Recently, cups manufactured from various forms of highly cross-linked PE have demonstrated a promising wear resistance in the medium term.^6,7^

Clinical wear can be assessed by measuring the change in displacement vector between the femoral head and cup from a series of antero-posterior (AP) radiographic images over time. The difference between the displacement vector on a follow-up radiograph and that on the initial, reference radiograph gives the resultant wear vector. A complication of this technique is that the wear plane may not always be parallel to the radiographic plane at successive radiographic examinations due to variations in the patient’s pelvis orientation.

Using a computer generated ‘virtual radiographic laboratory’, Foss et al.^8^ studied the effects of variations in pelvis orientation on the virtual wear measurement of an acetabular cup and concluded that measurement errors were likely to be small. Most modern measurement systems use the duo-radiographic wear measurement technique^9,10^ in which wear is determined by comparing the two-dimensional (2D) displacement vector (usually between the cup and head centres) on a follow-up radiograph to that on a reference (e.g. postoperative) radiograph. However, in their study, each of the wear measurements was of a single displacement vector. In addition, their analysis was only relevant for standard cups in which the initial socket centre was coincident with the overall cup centre. Later simulated duo-radiographic studies by The et al.^11,12^ found that for offset cup designs in which the centres of the socket and cup were separated by several millimetres, appreciable measurement errors were produced by variations in pelvis orientation. They proposed a mathematical formula (Appendix 1) to correct the wear vector. However, their formula had two major deficiencies. First, it only corrected the component of the wear vector perpendicular to the long axis of the elliptical cup opening image: the component parallel to the long axis was not corrected. Second, the correction was based solely on the change in the measured anteversion caused by a change in pelvis orientation: it did not account for the overall change in cup orientation – anteversion, inclination and rotation about the polar axis.

In this article, the effect of variations in pelvis orientation on 2D radiographic wear measurements is examined mathematically. A formula is developed for correcting the complete wear vector, taking into account the overall change in cup orientation caused by a change in pelvis orientation.

## Method

### Definitions

The change in position of the femoral head relative to the acetabular cup is caused by both creep and wear, although wear usually predominates after several months. For convenience, this relative change in position will be referred to in terms of ‘wear penetration’ and ‘wear direction’.

Changes in pelvic orientation are usually defined in terms of pelvic tilt (forward flexion or backward extension about a transverse, medial–lateral axis) and pelvic rotation (about the longitudinal, inferior–superior axis). For the purposes of 2D cup wear measurement, variations in the pelvis rotation about the anterior–posterior axis are accounted for directly by constructing a baseline reference parallel to the inferior borders of the ischial tuberosities or the ‘tear drops’.^13^

Cup orientation is usually defined in terms of version and inclination. Radiographic or planar inclination is the acute angle between the pelvic reference line and a line parallel to the cup base in the radiographic plane.^14^ On standard AP radiographs, the long axis of the elliptical cup opening (or wire marker) is used for the latter (Figure 1). Radiographic version is defined as rotation about an axis parallel to cup base in the radiographic plane (i.e. parallel to the inclination line). On AP radiographs, version is determined from the ratio of the semi-axis lengths (a, b) of the elliptical opening or wire marker (version = sin^−1^(b/a)). When the cup opening faces anteriorly, the cup is said to be anteverted (retroverted otherwise). To avoid impingement problems, a surgeon always aims to place a cup in anteversion.

**Figure 1.**
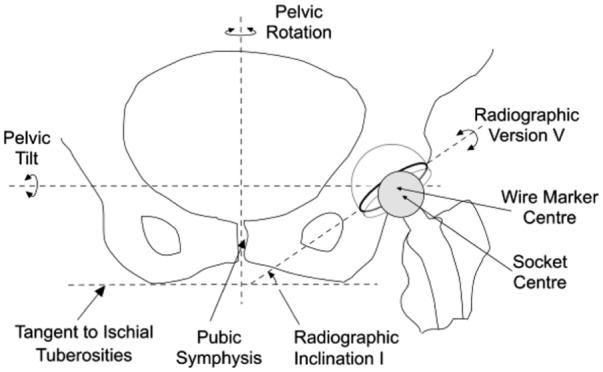
Diagram of the inferior aspect of a pelvis as viewed on an AP radiograph showing the proximal portion of a left total hip replacement. An offset, cemented cup is shown in which the base is extended so that the socket centre is infero-lateral to the cup centre.

For the complete 3D specification of cup orientation, it is also necessary to include rotation about an axis perpendicular to the cup base (i.e. the polar axis). This will be referred to simply as ‘polar rotation’. It can be determined by recording the co-ordinates of a landmark point on an AP image of the cup opening (e.g. a wire marker junction) or cup shell (e.g. a screw hole or liner locking ring) on serial radiographs (Figure 2). The co-ordinates of this point would then enable the rotational position relative to the extreme end of the elliptical opening to be calculated. In this way, changes in polar rotation can be determined mathematically.^15^

**Figure 2.**
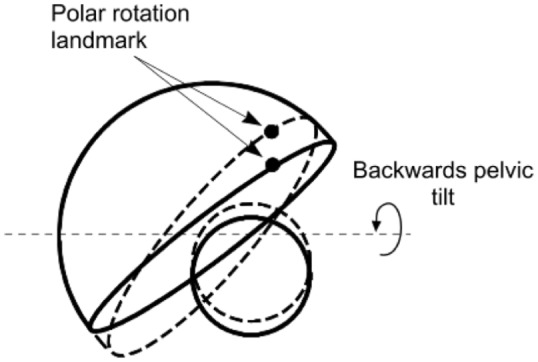
Following a backwards pelvic tilt (dashed lines), the orientation (version, inclination and polar rotation) of the cup changes together with the 2D position of the femoral head relative to the cup. Rotation of the cup about the polar axis is determined from the change in position of a chosen landmark relative to the lateral end of the cup opening. The changes have been exaggerated for clarity.

### Analysis

At the time of a follow-up radiograph, creep and wear would have changed the magnitude of the 2D displacement vector between the femoral head and cup. In addition, the 2D view of the pelvis might also have changed on that radiograph due to different patient positioning during the X-ray examination compared to that in the reference examination. The change in pelvis orientation on the radiograph would be accompanied by a change in the cup orientation and a change in the magnitude and direction of the 2D displacement vector measured on the radiograph (Figure 2).

The objective of this mathematical analysis is to recover the true magnitude and direction of the follow-up displacement vector as it would have been in the reference plane without a change in pelvis orientation. Its difference from the reference vector would then give the true wear vector. In the first part of the following analysis, therefore, the effect of a change in cup orientation on a displacement vector is considered. The resulting equations then enable the vector corresponding to the original cup orientation to be deduced. In the second part of the analysis, equations are developed to show the effect of a change in pelvic tilt on the measured cup orientation. In the third part of the analysis, the equations in the first two parts are used to correct a wear vector following pelvic tilt in two scenarios: when the wear plane is (1) parallel to the reference radiographic plane and (2) not parallel to that radiographic plane.

### The effect of a change in cup orientation on a displacement vector

The diagram in Figure 3(a) represents an AP radiographic view of an acetabular cup and femoral head in a right-hand axis system XYZ. The inclination ‘I’ (Figure 1) has been set to zero for convenience. The distance between the centres of the femoral head and cup is represented by the displacement vector OP of magnitude d and angle α to the long axis of the elliptical projection of the cup opening or wire marker. In Figure 3(a), the position of OP is initially in the plane of wear and also in the AP radiographic plane. The point Q_1_ represents a landmark point (such as a break in the circular wire marker or a circlip slot on the rim of a metal shell) which is used to determine a reference rotational position about the polar axis.

**Figure 3.**
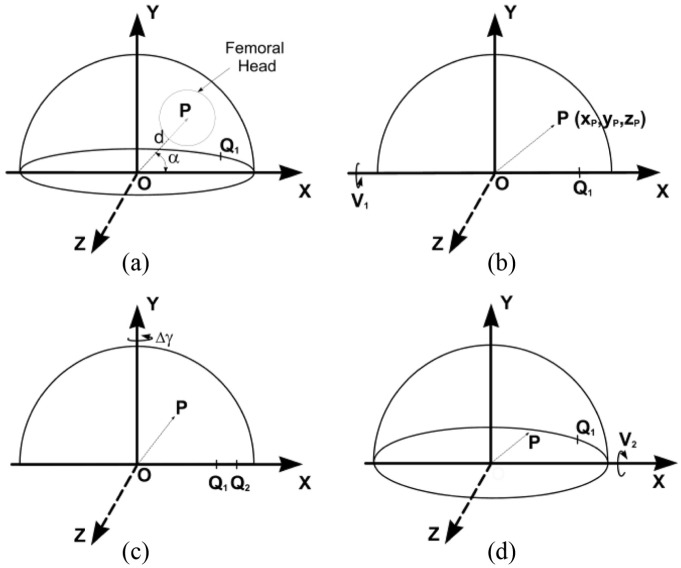
The effect of a change in cup orientation on a measured wear displacement vector, as viewed on an AP radiograph. The initial cup anteversion is V_1_ and its inclination I has been set to zero for convenience. (a) OP, a displacement vector between the measured centres of the cup and femoral head, initially has a magnitude d and an angle α to the inclination axis OX. The wear plane is parallel to the XY plane. Q_1_ is a chosen rotation landmark (here on the anterior rim of the hemispherical cup). (b) The cup has been rotated by V_1_ about the X-axis to bring the polar axis of the cup in line with the Y-axis. Point P moves to x_p_, y_p_, z_p_. (c) The effect on OP of rotation, Δγ, about the polar axis OY. Q_1_ moves to Q_2_. (d) The effect on OP of rotation (from the position in (b)) about the OX axis. The measured anteversion is now V_2_.

If the cup is rotated about the X-axis (Figure 3(b)) by an angle V_1_ (V_1_ is therefore positive here), the plane of the cup face becomes parallel to the XZ plane (i.e. zero version) and the co-ordinates of point P become $x_{p}=\cos\;\alpha$, $y_{p}=\sin\;\alpha \;\cos\;V_{1}$, $z_{p}=\sin\;\alpha \;\sin\;V_{1}$.

First, using rotation matrix algebra (Appendix 2, equation (8)), new XYZ values of point ‘P’ can be determined for a positive rotation, Δγ, about the Y-axis (Figure 3(c)). The value of Δγ can be determined from the measured changes in the position of points Q_1_ and Q_2_ (caused by the cup orientation change) relative to the lateral end of the cup opening/wire marker ellipse.^15^

Second, new XYZ values of point P can be determined (Appendix 2, equation (9)) for a *negative rotation* into anteversion, V_2_, about the X-axis (Figure 3(d)). Finally, equations for the magnitude $\bar{d}$ and direction $\bar{\alpha }$ of the 2D displacement vector resulting from the two rotations combined (polar rotation and anteversion) can be determined (Appendix 2, equations (10)–(13)). The original magnitude (d) and direction (α) of the displacement vector can then be deduced by rearrangement of the equations.

For the combined rotations

$$X=\cos\;\alpha \;\cos\;\Delta \gamma +\sin\;\alpha \;\sin\;V_{1}\;\sin\;\Delta \gamma$$

$$Y=\cos\;\alpha \;\sin\;V_{2}\;\sin\;\Delta \gamma +\sin\;\alpha \;\cos\;V_{1}\;\cos\;V_{2}-\sin\;\alpha \;\sin\;V_{1}\;\sin\;V_{2}\;\cos\;\Delta \gamma$$

d¯=X2+Y2andα¯=tan−1(YX)

- from which the original direction of OP can be calculated:

(1) $$\alpha =\tan^{-1}\left(\frac{\tan\;\bar{\alpha }\;\cos\;\Delta \gamma -\sin\;V_{2}\;\sin\;\Delta \gamma }{\cos\;V_{1}\;\cos\;V_{2}-\sin\;V_{1}\;\sin\;V_{2}\;\cos\;\Delta \gamma -\tan\;\bar{\alpha }\;\sin\;V_{1}\;\sin\;\Delta \gamma }\right)$$

and, using this value in X and Y, the original magnitude of OP can be calculated

(2) $$d=\frac{\bar{d}}{\sqrt{X^{2}+Y^{2}}}$$

### Effect of pelvic tilt on cup orientation

The effect of pelvic tilt on cup version and inclination (but not polar rotation) has previously been considered by others.^16,17^ In Figure 4(b), the cup anteversion has been set at –V_1_, which represents the version as measured on a reference radiograph (anteversion V_1_ is negative here – for a right-hand axis system). The displacement vector OP then points out of the radiographic plane and its 2D view (in the radiographic plane) becomes OP_1_. The components of vector OP then become $x=\cos\;V_{1}\;\sin\;I_{1}$; $y=\cos\;V_{1}\;\cos\;I_{1}$; $z=\sin\;V_{1}$.

**Figure 4.**
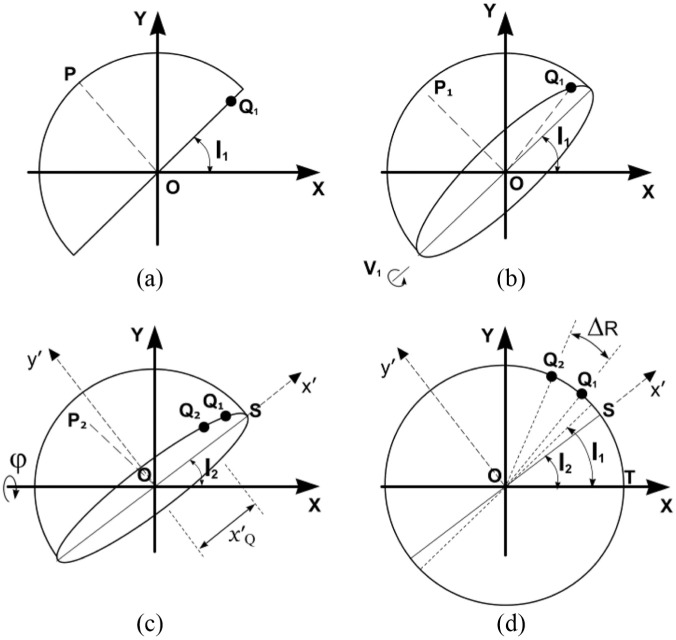
(a) Diagram representing an AP radiographic view of a left cup of unit radius in a global, right-hand axis system, XYZ. The cup inclination is set at an angle I_1_ to the X-axis – which is parallel to a line tangential to the ischial tuberosities (Figure 1). Version is initially zero. OP is a unit displacement vector in the direction of the polar axis of the cup. (b) The cup is anteverted to V_1_ causing point P to move out-of-plane to P_1_. Point Q_1_ is a designated rotation landmark (c) Forwards pelvic tilt φ applied about the X-axis causes a reduction in anteversion from V_1_ to V_2_, a reduction in inclination from I_1_ to I_2_, a rotation of Q_1_ to position Q_2_, and a change of the 2D view of OP_1_ to OP_2_. (d) This shows the circular view (from below) of the cup base in (c) after rotating the cup about the inclination axis, Ox′, by π/2 – V_2_. The line OQ_1_ has rotated to OQ_2_ in an anticlockwise direction through an angle ΔR.

After a pelvic tilt through an angle φ (Figure 4(c)), the cup inclination can be determined from the rotation matrix (Appendix 3, equations (14)–(16))

(3) $$I_{2}=\tan^{-1}\left(\frac{x}{y\;\cos\;\varphi -z\;\sin\;\varphi }\right)$$

and the cup version becomes

(4) $$V_{2}=\sin^{-1}\left(y\;\sin\;\varphi +z\;\cos\;\varphi \right)$$

The effect of pelvic tilt on acetabular cup rotation about its polar axis can now be considered. In Figure 4, point Q_1_ on the rim of the cup opening represents a landmark rotation point chosen on the reference radiograph. After pelvic tilt, the line OQ_1_ rotates through an angle ΔR as Q_1_ moves to position Q_2_ (Figure 4(d)). However, if the angle ΔR was substituted for Δγ in equations (1) and (2), the resulting values of d and α would pertain to a cup that was originally at an inclination I_2_. The effect of the change in inclination therefore has to be taken into account using Δγ as the polar rotation, where

(5) $$\Delta \gamma =\left(Q_{2}\hat{O}T-I_{2}\right)-\left(Q_{1}\hat{O}T-I_{1}\right)$$

In equation (5), each bracket represents the angle (γ_2_, γ_1_) corresponding to the position of the rotation landmark point relative to the lateral end of the cup opening on the follow-up and reference radiographs, respectively. If a rotation point is on the anterior of the cup (i.e. the upper rim or wire marker when in anteversion), the rotation point angle is measured in an anticlockwise direction for a *left* cup and is negative (right-hand axis system). If the rotation point is on the posterior of a *left* cup, the angle is positive. Here, therefore, the values of γ_1_ and γ_2_ are both negative, Δγ is negative and the left cup has internally rotated (Figure 4(d)). For measurements of a cup on the patient’s right side, a reflection of a cup on the left side can be used because it is conventional to measure the angles (inclination, displacement vectors) relative to the lateral side of the right cup opening axis (e.g. inclination = 45°, not 135°). The rotation point angles, γ_1_, γ_2_, should be measured from the lateral end of that axis but, in this case, they are positive in a clockwise direction if the rotation points are on the anterior rim (negative otherwise). A cup that has internally rotated (left or right hip) is associated with a reference cup inclination that is greater than the follow-up inclination (and vice versa) as in Figure 4(d).

For the purpose of the following error analysis calculations, the rotation point Q_1_ was set at the lateral end of the elliptical opening before pelvic tilt (Figure 4(b)). This simplified the calculation by making γ_1_ zero (equation (5)). OQ_1_ then represented a unit vector with components: $x=\cos\;I_{1}$, $y=\sin\;I_{1}$, z=0. Again, with an initial inclination I_1_ and anteversion V_1_, the distance $x\prime_{Q}$ (after pelvic tilt) was determined from X and Y in the rotation matrix (Appendix 3, equation (14))

$$x_{Q}\prime =X\;\cos\;I_{2}+Y\;\sin\;I_{2}$$

and so

$$x\prime_{Q}=\cos\;I_{1}\;\cos\;I_{2}+\sin\;I_{1}\;\cos\;\varphi \;\sin\;I_{2}$$

For the case where the rotation point is on the rim of the cup (radius R)

Δγ=cos−1(xQ′R)

For the purposes of the following error analysis, the cup is assumed to have a unit radius and, using I_2_ from equation (3)

(6) $$\Delta \gamma =\cos^{-1}\left(x\prime_{Q}\right)$$

The left cup has internally rotated and Δγ is, therefore, negative (Figure 3(c)). If the chosen rotation point is not on the cup rim/wire marker (i.e. not on the cup equator), the mathematics shown in Appendix 4 can be used.

### Error analysis

The value of α in equation (1) refers to the positive displacement vector direction shown in Figures 3 and 4. However, for the types of acetabular cup pertinent to this analysis, the femoral head centre would be below the centre of the cup opening or wire marker (Figure 5) and so the values of α would be negative. For cases where α was less than –π/2, equation (1) would give a positive value for α; therefore, π should be subtracted in order to make it negative.

**Figure 5.**
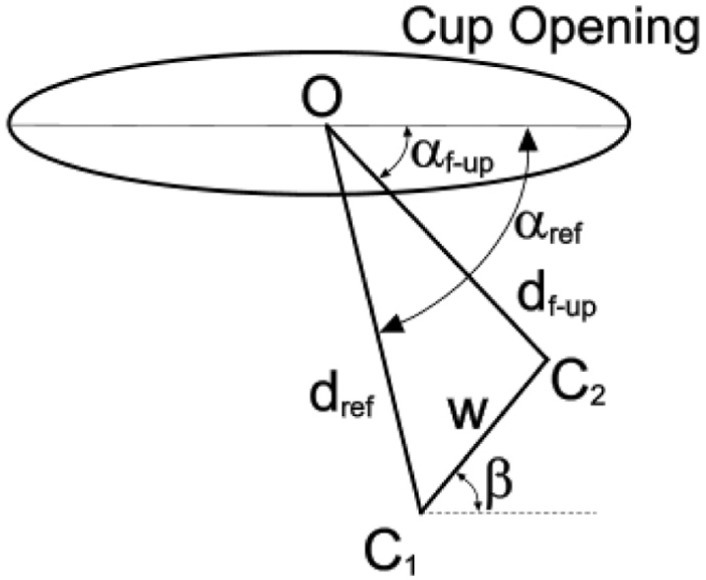
Geometrical representation of the opening of an offset cup in which the centre of the socket is below the wire marker/shell centre. The displacement vectors d_ref_ and d_f-up_ between the centre ‘O’ of the wire marker/shell and the centre of the femoral head (C_1_, C_2_, respectively) are measured on the reference and follow-up radiographs. Here, d_ref_ and d_f-up_ are in the radiographic, wear plane. The resulting wear vector ‘w’ is at angle β to the cup opening axis. For convenience, the inclination of the cup has been set at zero.

The cup design was assumed to be such that the centre of the socket was 4 mm below the centre of the cup opening or circular wire marker. In addition, the initial difference in sizes of the femoral head and cup socket (radial clearance) was taken into account. An arbitrary lateral shift of the femoral head of 0.106 mm (0.15 × cos(45)) was, therefore, assumed and so the angle of the reference displacement vector (α_ref_) was greater than –π/2, as shown in Figure 5.

The true penetration ‘w’ (due to wear and creep) was set at 0.3, 0.5, and 1.0 mm and different initial (reference) cup inclinations I_1_, anteversions V_1_ and penetration directions β were considered. In the first series of measurements, the reference displacement vector d_ref_ was assumed to be the immediate postoperative vector, and the follow-up vector d_f-up_ was determined relative to this in accordance with the wear penetration ‘w’ and the wear direction β. In the second series of measurements, the reference (wear plane) vector was considered to be in the position of d_f-up_ in Figure 5 (i.e. the vectors were swapped around so that d_f-up_ was the reference, wear plane vector, and d_ref_ the follow-up vector). The latter series was determined for comparison purposes because Thé et al.^11,12^ had chosen to carry out their measurements in this way. Using equations (3)–(5), the effects of pelvic tilt (ranging between ±20°^18–21^) on cup orientation and the displacement vectors were determined mathematically. The correction formulae (equations (1) and (2)) were then applied to the resulting ‘wear measurements’ and their efficacies were assessed as follows.

First, the reference displacement vector was assumed to be parallel to the wear plane. The magnitude and direction of the follow-up displacement vector ($\bar{d}$, $\bar{\alpha }$), after pelvic tilt, were calculated and were then verified with a 3D CAD system (DesignCAD 3D Max, www.imsidesign.com) using a circle to represent the cup opening (or wire marker) and a line to represent the displacement vector. Values of the uncorrected penetration magnitude and direction were calculated from the two displacement vectors. The magnitude and direction of the follow-up displacement vector were then corrected to their values (d, α) in the reference plane using equations (1) and (2) and the corrected penetration and direction were calculated. In addition, the 2D components of the reference and (uncorrected) follow-up vectors perpendicular to, and in line with, the cup opening/wire marker ellipse axis were used to calculate the wear penetration and direction using the method of Thé et al.^11,12^ (Appendix 1).

Second, the reference vector (reference plane) was set so that it was *not* parallel to the wear plane by applying, mathematically, a known ‘pelvic tilt’ to the original, in-plane, reference vector. Viewed above the reference plane, therefore, the reference displacement vector appeared shorter than it should have been. The follow-up displacement vector was also subjected to a different pelvic tilt, as above, but it was corrected back to the wrongly assumed reference wear plane. The resultant wear penetration and direction were again calculated.

## Results

Multiple data sets were created and analysed using a specifically created, Visual Basic, spreadsheet program (see Supplementary Material). Each data set contained 10 subsets determined from 10 wear directions (0°–90°). Tables 1 and 2 show selected examples of the effect of pelvic tilt (range ±20°) at the reference and follow-up stages on cup version, inclination, polar rotation and wear penetration/direction. Selection was based on a range of wear directions of ±20° relative to the ‘anatomical’ superior direction (lateral positive for a left cup) – a range that is generally representative of the wear direction.^22–24^ Accordingly, the wear direction β, relative to the cup opening axis, varied with the cup inclination. Wear penetrations of 0.3, 0.5 and 1.0 mm were considered (representing highly cross-linked PE wear and standard PE wear).

**Table 1.** 

Ref. cup inclination	Ref. pen. direction	Pelvic tilt		Cup version			Cup rot’n	Cup inclination		Uncorrected		Thé formula		This study	
		Ref.	Follow-up	Ref.	New ref.	Follow-up		New ref.	Follow-up	Pen.	Dir’n	Pen.	Dir’n	Pen.	Dir’n
True penetration 0.3 mm															
30	60	–	20	−10	–	7.4	−9.9	–	29.8	0.49	58.7	0.51	60.1	0.30	60.0
45	40	–	10	−10	–	−2.9	−7.1	–	44.2	0.38	36.1	0.41	41.9	0.30	40.0
50	20	–	−20	−20	–	−31.9	18.0	–	58.0	0.19	50.7	0.26	62.4	0.30	20.0
60	30	10	−10	−10	−4.9	−14.9	17.6	58.9	61.9	0.14	69.1	0.05	18.2	0.20	31.1
40	30	−10	−15	−20	−27.5	−31.1	4.0	42.9	44.9	0.24	42.7	0.18	11.0	0.33	29.1
55	30	10	−15	−30	−24.0	−37.7	24.5	50.9	63.7	0.48	14.7	0.61	39.9	0.17	30.1
True penetration 0.5 mm															
45	30	–	−10	−15	–	−21.9	7.6	–	47.4	0.40	39.7	0.32	18.8	0.50	30.0
60	30	–	5	−10	–	−7.5	−4.4	–	59.3	0.55	28.4	0.56	30.6	0.50	30.0
40	40	–	−15	−25	–	−36.0	11.9	–	46.1	0.35	75.9	0.1	−32.7	0.50	40.0
45	40	5	15	−20	−16.4	−9.1	−7.0	43.8	42.3	0.64	37.5	0.71	43.7	0.53	41.4
50	40	10	20	−15	−8.5	−1.8	−7.5	48.4	47.8	0.61	39.7	0.64	42.1	0.55	41.6
50	20	−10	−20	−15	−21.3	−27.1	8.9	52.6	56.2	0.35	29.5	0.31	0.4	0.55	19.3
True penetration 1.0 mm															
60	20	–	−20	−20	–	−28.8	19.8	–	68.3	0.54	26.9	0.48	−1.2	1.00	20.0
60	20	–	−20	−30	–	−38.2	22.1	–	72.5	0.34	36.3	0.30	−25.3	1.00	20.0
30	40	–	20	−30	–	−12.3	−10.1	–	26.3	1.29	39.1	1.50	48.1	1.00	40.0
60	30	−10	−20	−20	−24.7	−28.8	10.2	63.6	68.3	0.76	31.7	0.70	23.1	1.01	26.4
60	30	10	−20	−20	−14.8	−28.8	28.7	57.3	68.3	0.45	59.0	0.24	10.0	0.91	32.7
45	50	−10	15	−20	−26.9	−9.1	−18.6	48.2	42.3	1.29	39.4	1.49	47.8	0.86	47.7

Examples of the effect of a change in pelvic tilt between reference and follow-up radiographs on measured and corrected wear vectors. True (in the reference wear plane) wear penetrations of 0.3, 0.5 and 1.0 mm at different wear directions, β, are shown. The effect of wrongly assuming the wear plane was coincident with the reference radiographic plane was simulated by also tilting the reference plane (columns 3, 6, 9). The wear penetrations and directions corrected using equations (1) and (2) are shown together with corrections using the formula of Thé et al. Negative pelvic tilt is backwards. Negative version is anteversion. A left hip was assumed for the calculations. Values are in millimetres and degrees.

**Table 2.** 

Ref. cup inclination	Ref. pen. direction	Pelvic tilt		Cup version			Cup rot’n	Cup inclination		Uncorrected		Thé formula		This study	
		Ref.	Follow-up	Ref.	New ref.	Follow-up		New ref.	Follow-up	Pen.	Dir’n	Pen.	Dir’n	Pen.	Dir’n
True penetration 0.3 mm															
30	60	–	20	−10	–	7.4	−9.9	–	29.8	0.09	68.1	0.07	59.6	0.30	60.0
45	40	–	10	−10	–	−2.9	−7.1	–	44.2	0.22	47.8	0.18	36.1	0.30	40.0
50	20	–	−20	−20	–	−31.9	18.0	–	58.0	0.68	0.5	0.79	30.1	0.30	20.0
60	30	10	−10	−10	−4.9	−14.9	17.6	58.9	61.9	0.49	18.9	0.54	31.1	0.41	31.1
40	30	−10	−15	−20	−27.5	−31.1	4.0	42.9	44.9	0.37	14.6	0.42	32.4	0.26	23.5
55	30	10	−15	−30	−24.0	−37.7	24.5	50.9	63.7	0.99	8.5	1.19	34.5	0.45	35.7
True penetration 0.5 mm															
45	30	−	−10	−15	–	−21.9	7.6	–	47.4	0.61	21.6	0.68	34.0	0.50	30.0
60	30	–	5	−10	–	−7.5	−4.4	–	59.3	0.45	32.7	0.44	29.8	0.50	30.0
40	40	–	−15	−25	–	−36.0	11.9	–	46.1	0.74	19.5	0.97	44.0	0.50	40.0
45	40	5	15	−20	−16.4	−9.1	−7.0	43.8	42.3	0.34	50.0	0.27	35.6	0.46	40.7
50	40	10	20	−15	−8.5	−1.8	−7.5	48.4	47.8	0.35	45.9	0.32	41.0	0.42	41.6
50	20	−10	−20	−15	−21.3	−27.1	8.9	52.6	56.2	0.63	6.8	0.68	21.9	0.43	13.7
True penetration 1.0 mm															
60	20	–	−20	−20	–	−28.8	19.8	–	68.3	1.41	11.4	1.48	21.6	1.00	20.0
60	20	–	−20	−30	–	−38.2	22.1	–	72.5	1.62	9.0	1.71	20.4	1.00	20.0
30	40	–	20	−30	–	−12.3	−10.1	–	26.3	0.66	45.3	0.47	11.6	1.00	40.0
60	30	−10	−20	20	−24.7	−28.8	10.2	63.6	68.3	1.17	19.2	1.23	25.2	0.93	26.4
60	30	10	−20	−20	−14.8	−28.8	28.7	57.3	68.3	1.55	18.5	1.71	30.8	1.16	32.7
45	50	−10	15	−20	−26.9	−9.1	−18.6	48.2	42.3	0.72	68.9	0.41	50.6	1.19	46.0

This is the same as Table 1 except that the reference and follow-up displacement vectors (dref, df-up) have been swapped around. The signs of wear vector values have been changed accordingly. Note: the wear values are not expected to be the same as in Table 1 because the magnitude and direction of the reference and follow-up displacement vectors and their respective pelvic tilts have changed.

At all settings, wear vectors (penetration ‘w’ and direction β) were corrected exactly using equations (1) and (2) if the reference radiographic plane was parallel to the true wear plane. In contrast, if the wear vectors were left uncorrected, they were always erroneous and the absolute error increased with increasing reference version and inclination. Moreover, wear vectors corrected using the formula by Thé et al.^11,12^ were, in almost all cases, inferior to the uncorrected vectors. The only exceptions occurred when the wear direction was very low (i.e when the wear vector was almost parallel to the cup opening axis). In those cases, the effect of the wear correction was minimal because the perpendicular component of the vector (Appendix 1) was only small. As an example, using the settings shown in Table 1 (1 mm penetration, first row) in which the wear plane was correctly assumed, a backwards pelvic tilt of –20° reduced the ‘measured’ penetration to 0.54 mm (–46%) and increased the penetration direction by 6.9° (34.5%). Following correction using the formula of Thé et al.,^11,12^ these changes were even worse (–52% and –106%, respectively). However, using the formulae derived in this study (equations (1) and (2)), the wear penetration and direction were corrected exactly. Calculating the wear vectors after swapping the reference and follow-up displacement vectors – as suggested by Thé et al.^11,12^– produced no appreciable improvement to the results (Table 2).

The lower rows of each penetration section in Tables 1 and 2 show the effect of wrongly assuming that the reference radiographic plane was parallel to the wear plane. An ‘error’ range of ±10° for the difference between the true and assumed wear plane was considered. In many of these cases, it was still advantageous to correct the wear vectors using equations (1) and (2). For instance, for the settings shown in last row of the 0.5 mm penetration data of Table 1, the pelvis on the simulated reference radiograph was given a backwards tilt of –10° relative to the true wear plane while that on the follow-up radiograph was given a backwards tilt of –20°. With these settings, the uncorrected ‘measurements’ underestimated wear penetration by 30% and overestimated wear direction by 47.5%. Again, the formula of Thé et al.^11,12^ gave worse results: 38% and 98% underestimation, respectively. However, using equations (1) and (2) of this study, the errors were relatively small: 10% overestimation and 3.5% underestimation, respectively. Nevertheless, there were still cases where the uncorrected vectors were *less* erroneous than the corrected vectors. These cases were generally evident if the penetration was low, the inclination was low (<45°) and the wear direction was high (>45°) (Figure 6). However, because of the number of variables involved, it was not possible to establish specific ranges or limits that would encompass all mixtures of settings (software used in this study can be downloaded from the online Supplementary Material).

**Figure 6.**
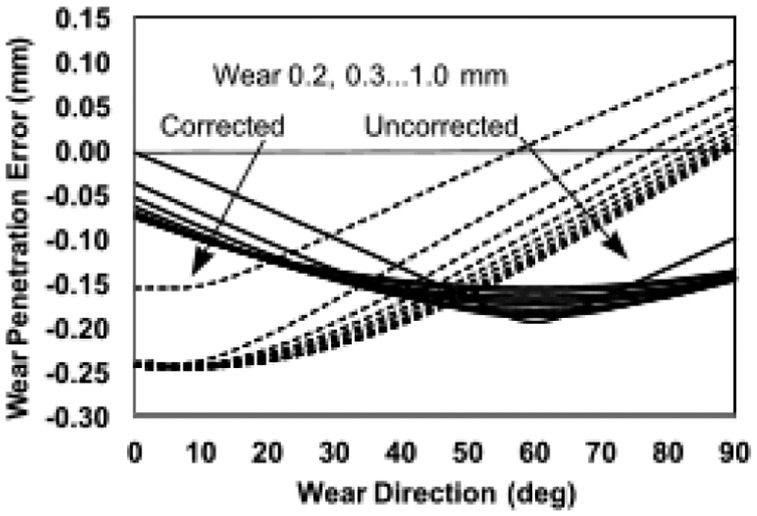
Chart showing the variation of corrected and uncorrected (dashed lines) wear penetration error with true wear direction (β) for a true penetration range of 0.2–1.0 mm (in arrow directions). Settings: reference tilt +10°, follow-up tilt –10°, inclination 30°, true anteversion –20°. Generally, the shape and intersections of the curves varied for different settings. Here, the corrected penetration error was less than about 50° and at mid-range penetrations.

## Discussion and conclusion

To measure 2D acetabular cup wear, the change in relative positions of the femoral head and cup images is measured from serial, AP radiographic images. Accurate measurement of this positional change depends upon a standardised view of the patient’s pelvis at each X-ray examination – which is impossible to achieve in a busy clinical environment. Variations in pelvis orientation are therefore inevitable. These variations are particularly problematic when measuring wear of offset cups, but this study has provided a means of correcting the wear for such variations (equations (1) and (2)).

An offset cup provides the surgeon with the option of adjusting the centre of rotation of the hip prosthesis close to the anatomical centre in order to restitute the hip kinematic and dynamic characteristics.^25^ The disadvantage of the offset from a wear measurement point of view is that it necessarily entails relatively large displacement vectors between the femoral head and cup centre. When the pelvis orientation changes, a ‘lever’ effect is created by these large displacement vectors and this magnifies the concomitant change in position of the projected image of the head relative to the cup. For example, if the socket centre were 4 mm below the cup centre, a backwards, –20° tilt would reduce a true wear of 0.4 mm by approximately 0.15 mm (V_1_ = –20°, I_1_ = 45°, β = 30°). In contrast, standard acetabular cups (i.e. with no offset) are usually designed so that the centres of the cup (or wire marker) and the PE socket are coincident. With these cups, the magnitude of the displacement vectors would be relatively small (dependent upon the wear magnitude) and pelvic orientation changes would cause only a small change in the measured wear. A true wear penetration of 0.4 mm, for instance, would be reduced by approximately 0.02 mm (same settings as above), which is below the accuracy of the best radiographic wear measurement systems.^26^ In their theoretical study of a standard cup, Foss et al.^8^ also concluded that wear measurement errors caused by changes in pelvis orientation were likely to be small compared to other error sources. Notably, though, they did not determine wear from two, sequential displacement vectors (reference and follow-up) using the duo-radiographic technique.^9,10^

For exemplification purposes, the simulated changes in the displacement vectors in this study have been produced by simulated changes in pelvic tilt. In clinical radiographs, combinations of pelvic tilt and pelvic rotation would be more likely, although pelvic tilt would usually predominate. This would not affect the use of the correction formulae because they depend solely on changes in the measured cup orientation – not on how those changes were brought about. This also applies when considering the effect of X-ray beam offset.^15^ In most cases, acetabular cup wear measurements are taken from AP radiographs of the full pelvis, and the central beam of the X-rays is targeted in the region of the pubic symphysis – not at the hip prosthesis (Figure 1). This introduces a small change in the recorded cup orientation on the reference and follow-up radiographs (anteversion 1°–2°, inclination 1°, polar rotation 5°–6°, approximately).^15^ However, since the correction formulae depend upon the *change* in the measured cup orientation, the wear vector would still be corrected to the reference plane – even if (through accidental misplacement by the radiographer) the location of the X-ray target point on a follow-up radiograph was different to that on the reference radiograph.

The derivation of the correction formulae is based on the assumption that the plane of the reference radiograph is parallel to the wear plane. Since this assumption cannot be guaranteed, it might be considered to be a major limitation of the formulae. However, the error analysis (Tables 1 and 2) showed that for many cases where the simulated plane of the reference radiograph was tilted (pelvic tilt) relative to the true wear plane, it could still be advantageous to correct the wear vector. This was not always the case, though, because some combinations of cup orientation, tilt and wear magnitude/direction resulted in the uncorrected wear vector being the better option. To a large extent, the attribution of an unknown tilted wear plane is a matter of judgement: one needs to know the sort of tolerance required for a given mixture of measurement settings before deciding whether to use the uncorrected values. To help with this, the spreadsheet program used for this analysis is available in the Supplementary Material. It should be acknowledged that the plane of the reference radiograph in this analysis did not include simulated pelvic rotation in addition to tilt, but the effect of this is probably minimal because pelvic rotation is relatively small (few degrees) compared to tilt.^17^

This study has revealed two other noteworthy features relating to the effect of a change in pelvis orientation. First, contrary to intuitive expectations, changes in pelvis orientation can cause an *increase* in the measured (uncorrected) wear penetration (Tables 1 and 2). Second, the correction formula proposed by Thé et al.^11,12^ is ineffective. In almost all cases in the present study, their correction was worse than the uncorrected measurements. Again, this is because the total change in cup orientation was not accounted for in their formula.

In conclusion, for cases where follow-up wear penetration is relatively large or for cup designs in which the cup and socket centres are not coincident, the method proposed in this study can provide a useful correction even if the exact reference plane of wear is not known. It should find a place in correcting data points and smoothing-out wear measurement graphs where variations in wear are associated with obvious changes in pelvis or cup orientation.

## Figures and Tables

**Figure 7.**
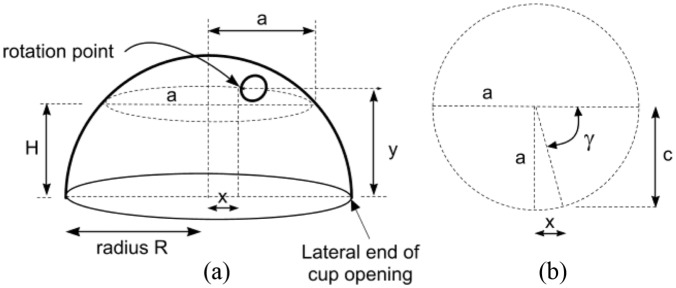
(a) The rotation point landmark is chosen to be at the edge of screw hole in the anterior surface of the shell image. (b) Circular view (from above the cup dome) of the cup section containing the rotation point landmark.

## Appendix 1

### Formula according to Thé et al.^11,12^

The following notation is similar to that described in Figure 5. The displacement vector correction method proposed by Thé et al.^11,12^ can only correct the component of the ‘tilted’ vector d_f-up_ which was perpendicular to the long axis of the elliptical cup opening or wire marker. The corresponding vector component along the ellipse axis was not corrected.

Perpendicular component, w_perp_

$$w_{\mathrm{perp}}=d_{\mathrm{ref}}\;\sin\;\alpha_{\mathrm{ref}}-d_{f\mathrm{up}}\;\sin\;\alpha_{f\mathrm{up}}\;\cos\;V_{1}/\cos\;V_{2}$$

Component parallel to the ellipse long axis, w_axis_

$$w_{\mathrm{axis}}=d_{\mathrm{fup}}\;\cos\;\alpha_{f\mathrm{up}}-d_{\mathrm{ref}}\;\cos\;\alpha_{\mathrm{ref}}$$

The resultant, w, of the above two components is then given by

(7) $$w=\sqrt{w_{\mathrm{perp}}^{2}+w_{\mathrm{axis}}^{2}}$$

## Appendix 2

### Equation derivations for orientation induced changes in the displacement vector

(8) $$\left(\begin{array}{c} X\\ Y\\ Z \end{array}\right)=\left(\begin{array}{ccc} \cos\;\Delta \gamma & 0 & \sin\;\Delta \gamma \\ 0 & 1 & 0\\ -\sin\;\Delta \gamma & 0 & \cos\;\Delta \gamma \end{array}\right)\left(\begin{array}{c} x_{p}\\ y_{p}\\ z_{p} \end{array}\right)$$

(9) $$\left(\begin{array}{c} X\\ Y\\ Z \end{array}\right)=\left(\begin{array}{ccc} 1 & 0 & 0\\ 0 & \cos\;V_{2} & -\sin\;V_{2}\\ 0 & \sin\;V_{2} & \cos\;V_{2} \end{array}\right)\left(\begin{array}{c} x_{p}\\ y_{p}\\ z_{p} \end{array}\right)$$

(10) $$\left(\begin{array}{c} X\\ Y\\ Z \end{array}\right)=\left(\begin{array}{ccc} 1 & 0 & 0\\ 0 & \cos\;V_{2} & -\sin\;V_{2}\\ 0 & \sin\;V_{2} & \cos\;V_{2} \end{array}\right)\left(\begin{array}{ccc} \cos\;\Delta \gamma & 0 & \sin\;\Delta \gamma \\ 0 & 1 & 0\\ -\sin\;\Delta \gamma & 0 & \cos\;\Delta \gamma \end{array}\right)\left(\begin{array}{c} x_{p}\\ y_{p}\\ z_{p} \end{array}\right)$$

which becomes

(11) $$\left(\begin{array}{c} X\\ Y\\ Z \end{array}\right)=\left(\begin{array}{ccc} \cos\;\Delta \gamma & 0 & \sin\;\Delta \gamma \\ \sin\;V_{2}\;\sin\;\Delta \gamma & \cos\;V_{2} & -\sin\;V_{2}\;\cos\;\Delta \gamma \\ -\sin\;\Delta \gamma \;\cos\;V_{2} & \sin\;V_{2} & \cos\;\Delta \gamma \;\cos\;V_{2} \end{array}\right)\left(\begin{array}{c} x_{p}\\ y_{p}\\ z_{p} \end{array}\right)$$

and so

(12) $$X=x_{p}\;\cos\;\Delta \gamma +z_{p}\;\sin\;\Delta \gamma$$

(13) $$Y=x_{p}\;\sin\;V_{2}\;\sin\;\Delta \gamma +y_{p}\;\cos\;V_{2}-z_{p}\;\sin\;V_{2}\;\cos\;\Delta \gamma$$

## Appendix 3

### Equation derivations for pelvic tilt induced changes in cup orientation

(14) $$\left(\begin{array}{c} X\\ Y\\ Z \end{array}\right)=\left(\begin{array}{ccc} 1 & 0 & 0\\ 0 & \cos\;\varphi & -\sin\;\varphi \\ 0 & \sin\;\varphi & \cos\;\varphi \end{array}\right)\left(\begin{array}{c} x\\ y\\ z \end{array}\right)$$

Thus, cup inclination I_2_ and version V_2_ after pelvic tilt through angle φ are given by

(15) $$I_{2}=\tan^{-1}\left(X/Y\right)$$

(16) $$V_{2}=\sin^{-1}(Z)$$

## Appendix 4

### Calculation of the polar rotation angle for any landmark position

For uncemented cups, the chosen rotation point landmark does not need to be on the rim of the cup opening. Figure 7(a) shows a screw hole in the anterior surface of a cup shell. The point of contact of a tangent to the screw hole, perpendicular to the cup opening axis, has been chosen as a landmark to set a rotation position. The radius of the shell is R, and its anteversion angle is V.

The dashed ellipse is parallel to the cup opening and passes through the landmark point. The point is at a distance x from the centreline of the cup image and a distance y perpendicular to the cup opening axis, where

(17) $$y=\cos\;V\sqrt{R^{2}-a^{2}}+\sin\;V\sqrt{a^{2}-x^{2}}$$

If the cup is retroverted, the last term in the equation should be subtracted instead of added. Equation (17) can be solved for ‘a’ using a numerical method (e.g. Newton–Raphson).

The rotation point angle is then given by

$$\gamma =\cos^{-1}\left(\frac{x}{a}\right)$$
